# BioSAXS Sample Changer: a robotic sample changer for rapid and reliable high-throughput X-ray solution scattering experiments

**DOI:** 10.1107/S1399004714026959

**Published:** 2015-01-01

**Authors:** Adam Round, Franck Felisaz, Lukas Fodinger, Alexandre Gobbo, Julien Huet, Cyril Villard, Clement E. Blanchet, Petra Pernot, Sean McSweeney, Manfred Roessle, Dmitri I. Svergun, Florent Cipriani

**Affiliations:** a European Molecular Biology Laboratory, Grenoble Outstation, 71 Avenue des Martyrs, CS 90181, 38042 Grenoble, France; b Unit for Virus Host Cell Interactions, Université Grenoble Alpes–EMBL–CNRS, 71 Avenue des Martyrs, CS 90181, 38042 Grenoble, France; c European Molecular Biology Laboratory, Hamburg Outstation, EMBL c/o DESY, Notkestrasse 85, 22603 Hamburg, Germany; d ESRF, 6 Rue Jules Horowitz, 38000 Grenoble, France

**Keywords:** small-angle X-ray scattering, BioSAXS Sample Changer, high-throughput, automation

## Abstract

Small-angle X-ray scattering (SAXS) of macromolecules in solution is in increasing demand by an ever more diverse research community, both academic and industrial. To better serve user needs, and to allow automated and high-throughput operation, a sample changer (BioSAXS Sample Changer) that is able to perform unattended measurements of up to several hundred samples per day has been developed. The Sample Changer is able to handle and expose sample volumes of down to 5 µl with a measurement/cleaning cycle of under 1 min. The samples are stored in standard 96-well plates and the data are collected in a vacuum-mounted capillary with automated positioning of the solution in the X-ray beam. Fast and efficient capillary cleaning avoids cross-contamination and ensures reproducibility of the measurements. Independent temperature control for the well storage and for the measurement capillary allows the samples to be kept cool while still collecting data at physiological temperatures. The Sample Changer has been installed at three major third-generation synchrotrons: on the BM29 beamline at the European Synchrotron Radiation Facility (ESRF), the P12 beamline at the PETRA-III synchrotron (EMBL@PETRA-III) and the I22/B21 beamlines at Diamond Light Source, with the latter being the first commercial unit supplied by Bruker ASC.

## Abstract 2

A robotic sample changer for solution X-ray scattering experiments optimized for speed and to use the minimum amount of material has been developed. This system is now in routine use at three high-brilliance European synchrotron sites, each capable of several hundred measurements per day.

## 1. Introduction   

Rigorous and accurate data collection for any experimental technique can be tedious, laborious work. Repetitive yet consistent and reproducible measurements are required in order to ensure the highest data quality, and these tasks must be automated to ensure the required stability. Small-angle X-ray scattering (SAXS), a structural method providing low-resolution information on macromolecules in solution, has experienced a dramatic increase in popularity in the structural biology community during the last decade. The improvements in SAXS instruments and in data-interpretation methods have attracted numerous new users and have made it necessary to automate the measurement process. The first robotized sample changer was developed (Round *et al.*, 2008 ▶) by the EMBL Hamburg Outstation and the Fraunhofer Institute (Stuttgart) in the frame of the EU-funded SAXIER project (http://www.saxier.org). The robot was designed to accomplish all of the necessary steps for sample handling to enable fully automated data acquisition according to the standard protocol in use at the EMBL X33 beamline (DESY DORIS-3 storage ring in Hamburg) for measurements of macromolecular solutions. The standard protocol for biological SAXS measurements requires the loading of a sequence of protein solutions with their matched buffers (with the latter measured before and after each sample). The measured buffer scattering should be subtracted from that of the protein solute and the two measurements must have the same instrumental background. This fact necessitates the use of the same measurement cell, which has to be cleaned and dried in between all measurements. The robot which performs all of the required actions was installed at the X33 beamline in September 2007 and rapidly demonstrated the advantages of automated operation. The workflow not only became much faster but the cleaning was also more thorough and reliable, leading to better background subtractions and increasing the data quality and confidence. The robot was rapidly adopted as the standard equipment, which increased the operational efficiency, freeing the time of the user groups on the experiment while maximizing the number of samples processed. Usage of the robot also minimized lost time, as the failure rate of sample loading was found to be less than 0.5%.

At the SOLEIL synchrotron, a system that combines an auto-sampler robot and online high-pressure liquid chromatography (HPLC) was developed for the SWING beamline (David & Pérez, 2009 ▶). In the sample-changer mode, samples of down to a few microlitres in volume are exposed in a glass capillary at controlled temperature, with a turnover of about 4 min. In the HPLC mode, an initially polydisperse sample can be separated into each of its components before immediate processing.

At the Advanced Light Source in Berkeley, the SIBYLS beamline was equipped with a robotized sample changer based on a Hamilton pipetting robot (Classen *et al.*, 2013 ▶). Solutions prepared in 96-well plates are transferred to open exposure cells with mica windows, where samples are exposed in a controlled atmosphere with a 5 min turnover.

The continuous advances made at third-generation synchrotrons in increasing the intensity of X-ray beams combined with the introduction of fast-readout single-photon counting hybrid silicon pixel detectors has further increased the need to reduce the sample-handling overhead. With the advent of dedicated BioSAXS beamlines being built at the ESRF and at the PETRA-III storage ring in Hamburg, a trilateral collaborative project was initiated between the EMBL Grenoble and Hamburg outstations and the ESRF to design and build systems that are able to process several hundred measurements per day while minimizing the volume of sample used per experiment.

Additionally, reliable and robust operation had to be ensured together with the provision of feedback on the status of the machine and of the ongoing procedure, necessitating the addition of sensing for collisions, the liquid level in the sample wells and video feedback to verify sample loading, positioning and cell drying. Below, the new Sample Changer, its operation and its implementation at the beamlines in Grenoble and Hamburg are described in detail.

## 2. Objectives and main concept   

**Figure 1:**
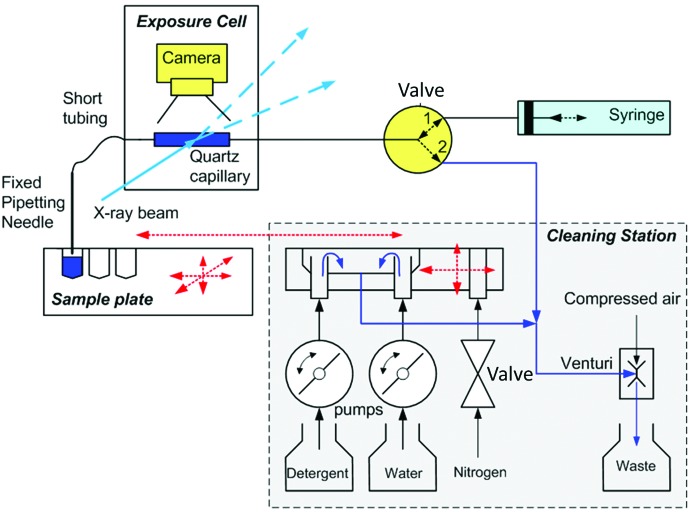
Schematic diagram of the Sample Changer. The sample plate mounted on a *XYZ* table is moved to the pipetting needle. Solution in a well is loaded in the exposure capillary by suction, aspirated by the syringe pump through the valve set in position 1. The syringe pump is also used to flow the solution in the capillary during exposure to X-rays. After exposure, the solution can be recuperated back into a sample plate. The cleaning station moves to the pipetting needle and the valve is set in position 2. The solution path and the outside of the pipetting needle are successively washed, rinsed and dried in three cleaning wells, with the exhaust flows terminating in a waste container aspirated by a Venturi pump. Both the sample-positioning and cleaning operations are controlled using a camera.

The objective of the project was to make the best use of the small and intense beams anticipated at the high-brilliance EMBL@PETRA-III and ESRF BioSAXS beamlines by measuring sample volumes of down to 5 µl and automatically processing several hundred samples stored in SBS Microplates in less than 1 min cycle time per sample. Another aim was to integrate in-line sample-concentration measurement and liquid-handling capacities to allow sample optimization directly in the sample changer. The key concept to achieve low sample volume and high sample turnover was to minimize the length of the fluidic path, using an architecture in which the exposure cell is connected to a fixed pipetting needle with very short tubing and the sample wells are moved towards the needle. The selected sample is aspirated from the back of the exposure cell with a syringe (Fig. 1 ▶). Minimizing the tube length not only reduced sample-loading times but also minimized the cell-cleaning time by reducing the surface area and thus significantly speeding up the drying.

Measurements of macromolecular solutions at high-brilliance beamlines must involve a flow of the sample through the beam path to minimize radiation damage. Effective use of the minimal sample volumes can be achieved by exposing the largest possible portion of the sample flowing through the X-ray beam during the measurement. Further, to ensure the optimal signal-to-noise ratio, the exposure cell should be placed in vacuum and its thickness appropriately selected. The value of 10^13^ photons s^−1^ focused in 0.3 mm (h) × 0.1 mm (v) was retained as typical beam characteristics for the target beamlines. For the useful energy range [8 keV (λ = 1.55 Å) at the EMBL X33 beamline and 13.3 keV (λ = 0.93 Å) at the ESRF ID14-EH3 beamline], the optimal sample thickness was between 1 and 1.7 mm. An ideal sample compartment would be in a thin-walled rectangular tube of the desired depth and a height corresponding to the vertical dimension of the beam. However, for practical reasons (mechanical resistance to vacuum) we opted for a 1.8 mm diameter thin-walled quartz capillary set horizontally in the X-ray beam.

Different capillary diameters can be selected to optimally use the range of X-ray energies available at the ESRF BM29 and PETRA-III P12 beamlines: 1 mm diameter for lower energies [below 10 keV (λ = 1.24 Å)] and 1.8 mm for higher energies [above 10 keV (λ = 1.24 Å)]. As the beam size is smaller than the vertical height of the capillary the parasitic scattering from the surface is minimized, but this comes at the expense of sample volume which does not interact with the beam. The advantage of this approach is that if small air bubbles appear at the top of the capillary out of the beam path they do not interfere with measurement.

## 3. Description of the Sample Changer   

The BioSAXS Sample Changer is designed to automatically expose micro-volumes of solution stored in SBS Microplates (Society for Biomolecular Screening, ANSI/SBS 1-2004) to X-rays. As little as 5 µl of solution can be transferred in a vacuum-mounted quartz capillary. After exposure, the fluid path is cleaned and dried automatically. The liquid handling of the Sample Changer can also be used to transfer (by pipetting) microlitre volumes from one selected well of the SBS Microplate to another, enabling dilutions or additions to initiate reactions remotely. The pipetting, mixing and sample-loading features can be used in combination with an in-line spectrometer for verification of sample concentration. The machine can be operated from a graphical user interface or fully controlled remotely from a client program such as the beamline-control software. The instrument comprises four subunits: (i) the Sample Exposure Unit, (ii) the Sample Changer Unit, (iii) the Fluid Management Unit and (iv) the Control Unit.

**Figure 2:**
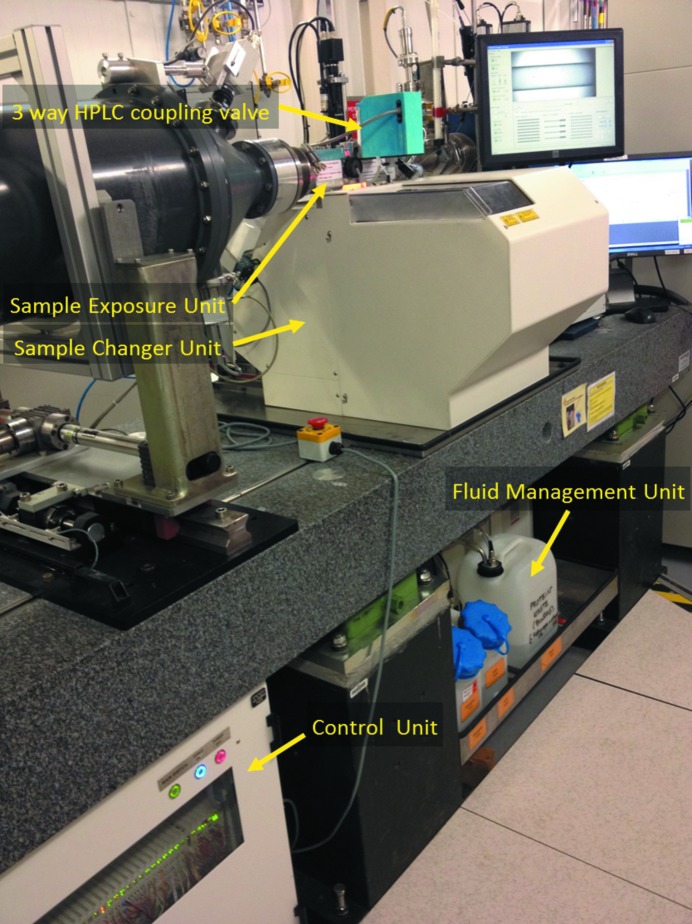
The BioSAXS Sample Changer installed on ESRF beamline BM29.

**Figure 3:**
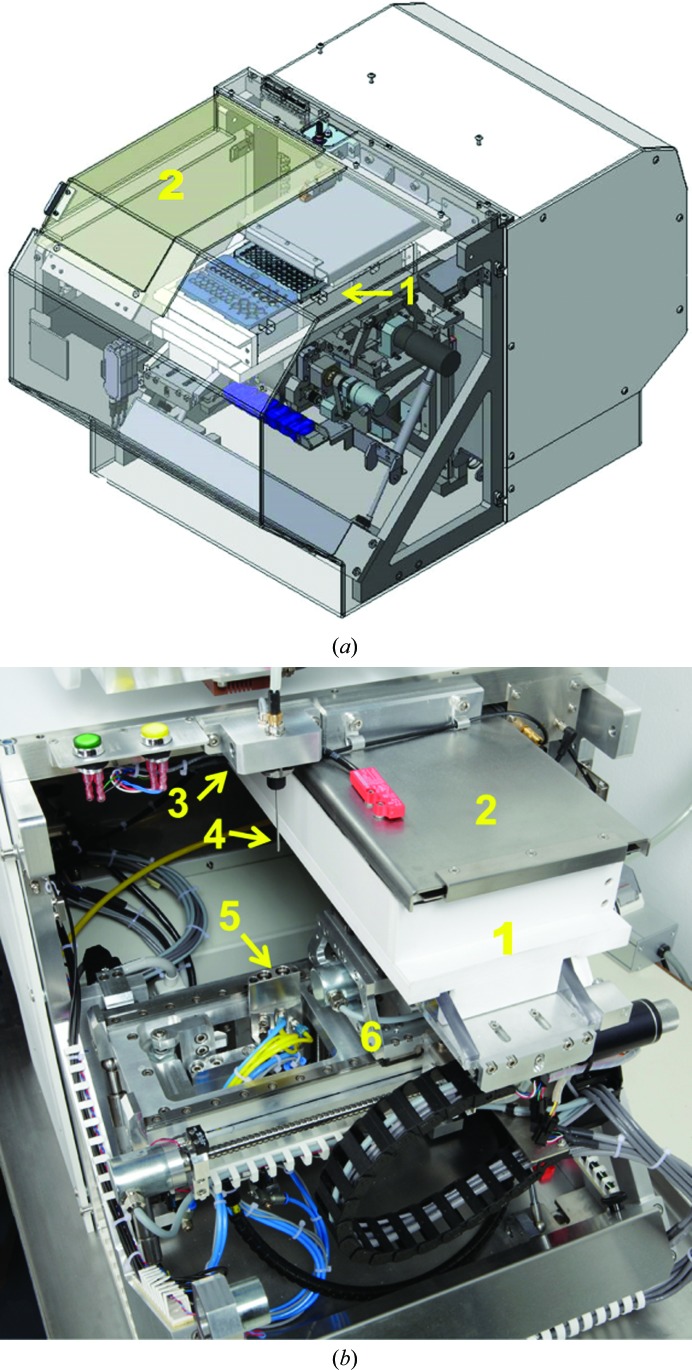
Sample Changer Unit. (*a*) Sample Storage Rack (1) in parked position (made visible as the fixed cover is shown as transparent in this image) and sliding port for sample access (2). (*b*) Internal view with Sample Storage Rack (1), insulated cover (2), Spectrophotometer Pod (3), Pipetting Needle (4), Cleaning Station (5) and *XYZ* table (6).

The Sample Changer Unit (Fig. 2 ▶) moves any selected sample to the Pipetting Needle of the Sample Exposure Unit automatically. It is composed of a thermo-regulated Sample Storage Rack mounted on an *XYZ* stage. Three slots in the Sample Storage Rack can receive different sample holders *via* specific barcoded mechanical interfaces. Interfaces are available for different SBS Microplates, strips of wells and individual wells. To limit sample evaporation and to ensure a precise storage temperature, the Sample Storage Rack is parked under an insulated cover unless a sample-transfer or pipetting operation occurs. All of the samples in the Sample Storage Rack are at the same temperature, which is defined from the Graphical User Interface of the machine or remotely by a host program. When switched on, the machine automatically identifies the types of sample-holder interfaces installed *via* the barcodes and reconfigures accordingly to the corresponding sample-holder topologies. The sample holders can be installed or removed by opening the top cover of the Sample Changer Unit that gives access to the Sample Storage Rack. Once loaded with samples, the machine checks that the sample holders are properly inserted, checks the straightness of the Pipetting Needle and optionally reads the barcodes of the sample holders for further tracking. The Sample Changer Unit hosts the Pipetting Needle, the Spectrophotometer Pod and the Cleaning Station (Fig. 3 ▶).

The Sample Storage Rack can receive three barcoded mechanical interfaces for (i) 96-well SBS Microplates (Greiner Bio-One, catalogue No. 651 201; http://www.greinerbioone.com); (ii) strips or individual wells, including four strips of 8 × 200 µl PCR wells (Greiner Bio-One, catalogue No. 673201) for samples plus 12 individual 1.5 ml PCR wells for buffer solutions (Eppendorf; catalogue No. 0030 120.086; http://www.eppendorf.com); (iii) V-shaped 96-well Thermo Fast Plates (ABgene; Thermo Scientific catalogue No. AB-0600; http://www.thermoscientificbio.com); and (iv) 96 Deep Wells SBS Microplates (ABgene; catalogue No. AB-0932). The SBS Microplates can optionally be barcoded. The temperature of the whole storage rack can be set between 4 and 40°C (±1°C).

**Figure 4:**
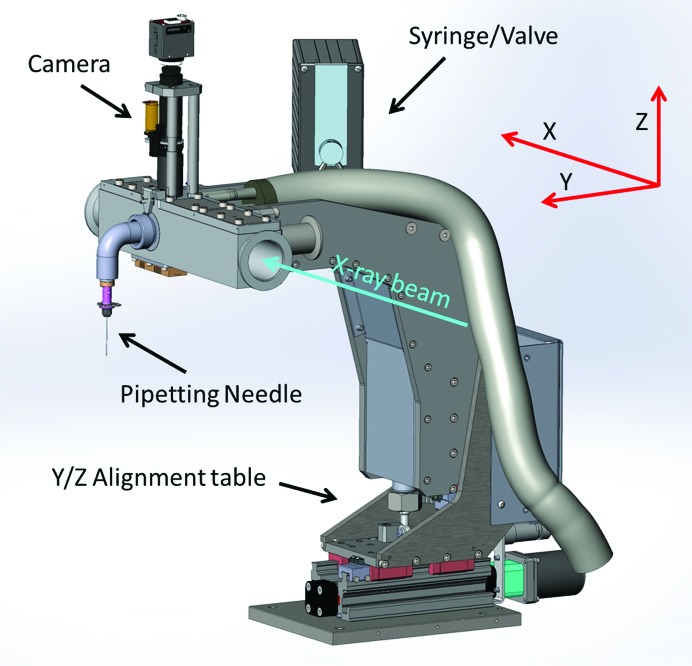
Sample Exposure Unit installed on the *YZ* alignment table, with the camera at the top (the orientation of the *XYZ* axes is shown in red).

**Figure 5:**
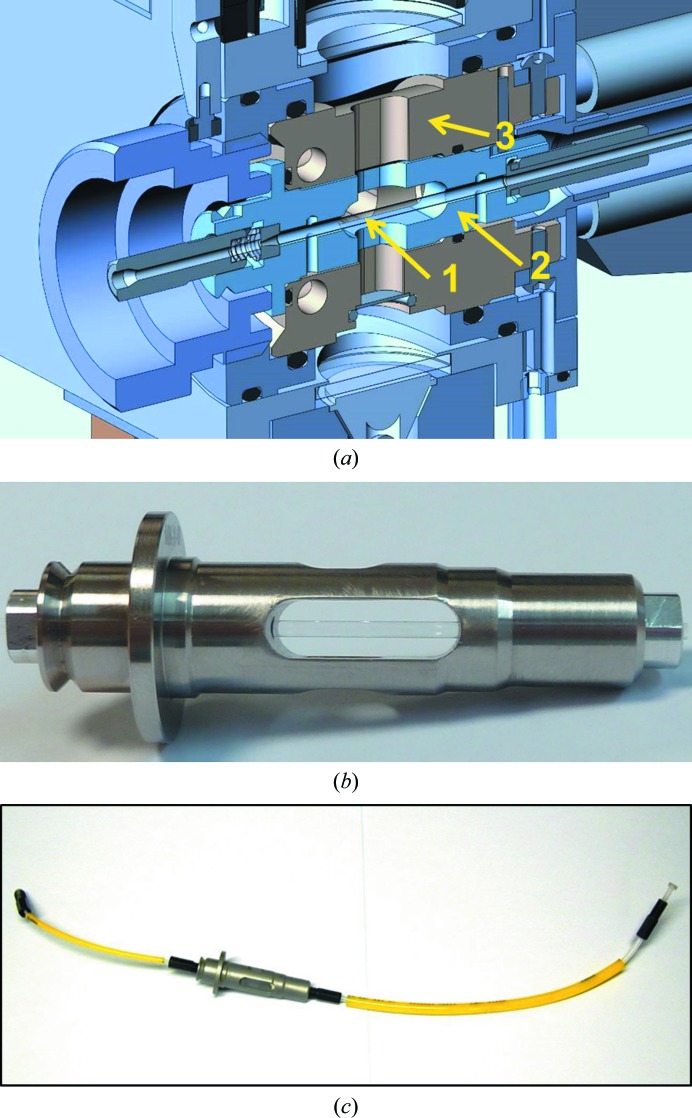
Exposure Cell. (*a*) Cross-section showing the Exposure Capillary (1), the Exposure Pod (2) and the Thermal Block (3). (*b*) Close-up view of the Exposure Pod. (*c*) Pre-assembled Exposure Pod with tubing and connectors, ready to be mounted in the Sample Exposure Unit. The left inlet corresponds to the short tube from the Pipetting Needle; the right outlet is the longer tube to the two-way valve which switches between syringe and waste.

The Sample Exposure Unit (Fig. 4 ▶) is composed of an Exposure Cell and a Pipetting System. The Exposure Cell is a vacuum-tight chamber that hosts the sample capillary. It is mounted on a *YZ* stage and should be placed in the beam path, preferably between two fast vacuum valves. The sample capillary is mounted in an easily replaceable stainless-steel Exposure Pod that is inserted in a thermal exchanger (Fig. 5 ▶). A video microscope and associated lighting system allows visualization of the sample capillary (Fig. 4 ▶). The Pipetting System sits close to the Exposure Cell. It is composed of a Pipetting Needle connected to one side of the Exposure Pod and a motorized syringe connected to the other side of the Exposure Pod *via* a two-way valve. For sample loading, the solution is aspirated from the sample well set under the Pipetting Needle to the sample capillary. Optionally, the sample can be recovered after exposure. The level of the solution in a well is detected using a sensor connected to the Pipetting Needle. This feature is used to minimize the contamination of the Pipetting Needle during solution transfers, to check that sufficient liquid is available in a well before a transfer into the Exposure Cell and to avoid liquid overflowing in the case of attempts to recover any solution into an already full well. When needed, the Pipetting Needle and the fluidic path are automatically cleaned. The Cleaning Station located in the SCU automatically washes (with cleaning solution), rinses (with distilled water) and dries (with dry air) the outer and inner side of the Pipetting Needle and the inner side of the exposure capillary and tubing. In this process, the two-way valve redirects the fluid path to the Fluid Management Unit (described below). The standard cleaning solution is a mixture of 2% Hellmanex III (a commercial detergent for cleaning quartz capillaries; http://www.hellma-analytics.com), 10% ethanol and 88% distilled water.

A Spectrophotometer Pod set in the fluid path between the Pipetting Needle and the Exposure Pod allows measurement of the concentration of the samples on demand or at sample-loading time. The Pipetting Needle and the Spectrophoto­meter Pod are physically located in the Sample Changer Unit.

**Figure 6:**
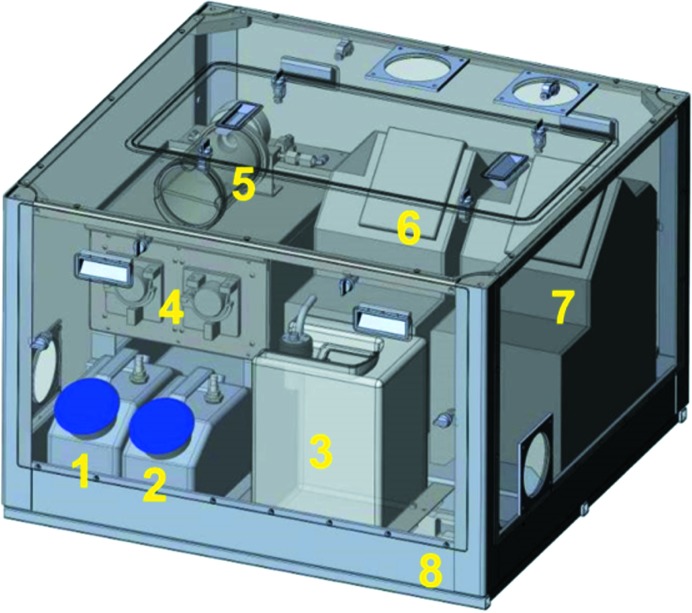
Schematic drawing of Fluid Management Unit with detergent reservoir (1), water tank (2), waste container (3), two Venturi pumps (4), exhaust filter (5), thermoregulated bath for Sample Changer Unit (6), thermoregulated bath for Sample Exposure Unit (7) and safety tray (8) in case of leaks.

The Fluid Management Unit houses a set of peristaltic pumps, aspiration devices, detergent and water supply reservoirs and a disposal container (Fig. 6 ▶) used by the Cleaning Station. The levels of two reservoirs and the disposal container are monitored and can be exported to the experiment control program to safely prepare an experiment. The Fluid Management Unit sits on a safety tray in case of fluid leaks, including those coming from the Sample Changer Unit. A fluid detector in the bath stops the machine to prevent any flooding. The Fluid Management Unit also hosts the two thermoregulated baths used to control the temperature of the Sample Storage Rack and Exposure Cell. The safety standards for the liquid waste integrate high redundancy with respect to leakage with double tubing on all fluid tubes and a unit for the collection of any eventual fluid leak.

The Control Unit is composed of an Electronic Rack, Windows PC and touchpad screen.

**Figure 7:**
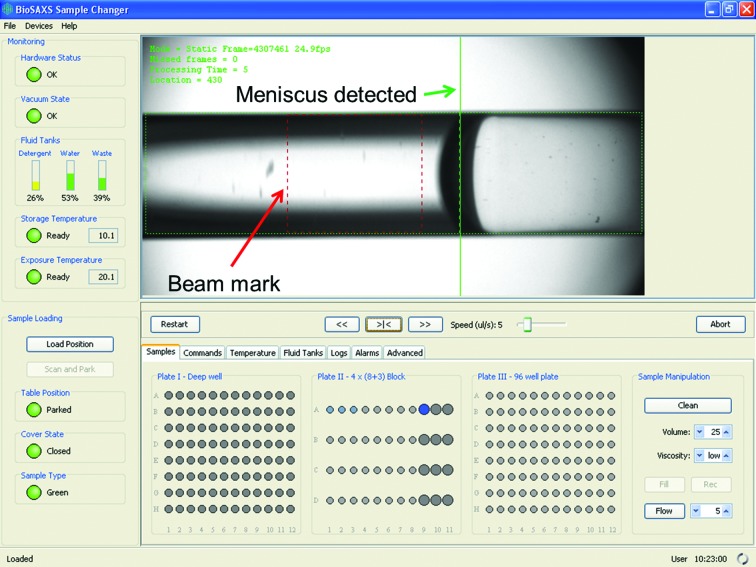
Screenshot of the Sample Changer control software with capillary view and various monitoring information as to which samples have already been loaded into the capillary (circles marked in dark blue), Fluid Tanks status *etc.* The Sample Loading panel (lower left) allows the Sample Storage Rack to be placed into the ‘Load Position’ or the ‘Scan and Park’ position needed for filling samples into the Sample Exposure Unit.

The control electronics are based on Beckhoff EtherCAT field bus modules (http://www.beckhoff.com) driven by TwinCAT real-time Programmable Logical Controller (PLC) and Numerical Control (NC) motion software. The control program of the machine runs on a Windows 7 PC. It was designed by focusing on its flexibility in order to simplify deployment and integration on different beamlines. The software is based on Java technology to execute functions implemented as Python scripts run by Jython. Each of the defined functionalities of the Sample Changer can be easily modified. This allows rapid adaptation to the local environment and the integration of new functionalities. The Graphical User Interface (GUI; Fig. 7 ▶) was designed to be operated easily through a touch panel located above the Sample Changer Unit. Typically, the GUI is also accessed from the beamline-control hutch through a Virtual Network Computing application or terminal duplication hardware. The whole functionality of the sample changer is available within the GUI and can also be accessed remotely through a server, thus enabling the automation of experiments *via* the beamline-control software.

The video feed is used with an automated image-processing algorithm to provide feedback on the meniscus position, which is used for sample loading, fixing the sample position (thermal drift) and active control during flow, as well as the drying process.

## 4. Operation of the Sample Changer   

### 4.1. Basic operations   

The samples are first introduced into the Sample Changer by clicking on the ‘Load Position’ button in the GUI (Fig. 7 ▶) and accessing the Sample Storage Rack by opening the top cover. After the sample plates or strips are installed in the storage rack the user must close the cover and press the ‘Scan and Park’ button, which starts a scan procedure that checks that the needle is straight and that the sample supports are well positioned. If no problems are detected then the robot parks the Sample Storage Rack under the cover and waits for commands.

The fundamental feature of the Sample Changer is to load a given volume of sample from a well into the Exposure Cell and to optionally flow the sample at a controlled speed while it is exposed to X-rays. In order to minimize sample losses in the tubing, the loading speed is determined by the viscosity of the sample. This can be set as low, medium or high from the GUI of the Sample Changer or by the beamline experiment-control software. The liquid is detected when reaching the Exposure Capillary by an image-processing algorithm (Fig. 7 ▶) and the syringe is stopped just after the meniscus (air–liquid interface) has passed the pre-defined beam position. A delay is applied if necessary before the sample reaches the pod, so that the sample achieves the requested exposure temperature once the load finishes. This delay is proportional to the difference between the storage and exposure temperatures. The machine has a volume-detection feature, which estimates the sample volume inside the well. A warning is reported if the available sample volume is smaller than the requested load volume. When entering a well, the needle is positioned at the minimal depth calculated to enable aspiration of the requested volume. In this way less liquid is lost on the needle walls and there is less needle contamination. The software can be configured to compensate for the losses along the tubing. In this case an extra volume is added to the requested volume.

During the data collection samples can be flowed to minimize radiation damage. The syringe speed is calculated as a function of the loaded volume and the given exposure time. The meniscus at the end of the sample is detected using the same image-processing algorithm as used for loading (Fig. 7 ▶) and the sample is stopped to prevent the solution exiting the beam.

After exposure, a sample can be recuperated to any given well. The transfer speed is determined based on the viscosity of the sample set from the GUI or provided by remote commands. Sample recuperation is automatically disabled for samples that are classed as harmful (*e.g.* yellow under ESRF safety regulations). The use of the Sample Changer is forbidden for samples classified as dangerous (*e.g.* red under ESRF safety regulations).

All surfaces of the Sample Exposure Unit which come into contact with a sample must be cleaned before any new sample is loaded. The cleaning operation washes, rinses and dries the needle, tubing and capillary. The timings are automatically set according to the viscosity. A real-time image-processing algorithm applied to the images of the capillary (Fig. 7 ▶) stops the drying procedure when no more droplets can be detected in the capillary.

### 4.2. Alternate sample input   

The Sample Exposure Unit can be coupled to a size-exclusion chromatography system to expose samples to X-rays immediately after purification. For this purpose, an optional three-way valve is installed between the Pipetting Needle and the Exposure Pod (Fig. 2 ▶). An online size-exclusion chromatography mode can be selected from the GUI of the robot. In this mode, the eluent coming from the size-exclusion chromatography system flows through the Exposure Cell when exposed to X-rays and is then directed to the waste of the Sample Changer. Only the control of the temperature of the Exposure Cell and storage remains active. All other functions are disabled for safety reasons.

The three-way valve enables automated switching between Sample Changer and size-exclusion chromatography modes and allows the sequencing of different kinds of experiments. Users can switch quickly and safely between measurement types, maximizing the efficiency of their experiments. Additionally, this valve offers protection to the system as the valve will return to a safe position (Sample Changer operation) if a vacuum error is detected. Consequently, eluent will not continue to flow through a broken capillary, for example.

## 5. Advanced features of the Sample Changer   

Whenever the pod is changed the machine parameters must be adjusted, such as the dead volume, the camera pixel:microlitre ratio, the capillary size and position on screen, and the focus positions for both capillary walls. These adjustments can be performed automatically using a Calibration routine, which is executed by clicking the appropriate GUI button.

When in static exposure mode, *i.e.* no flow option is used during sample exposure, the loaded sample tends to move, for example owing to a temperature gradient between the exposure cell and the end of the tubing or in the case of tiny leaks. In order to keep the sample in a fixed position, the control software offers the functionality of automated regulation. Meniscus movements that are detected are compensated with syringe pull/push actions to keep the meniscus in the desired place (Fig. 7 ▶), *i.e.* out of the beam mark. This feedback can be activated either from the GUI of the Sample Changer or through the experiment-control software.

The tubing from the needle to the pod is as short as possible in order to minimize losses. The tubing dead volume is typically around 100 µl. Larger diameter tubing can be used to handle larger sample volumes (up to 250 µl). Any modification of the tubing requires recalibration of the robot parameters using the auto-calibration procedure.

The robot includes an in-line spectrophotometer system that can be used for protein concentration measurements. A quartz capillary pod installed between the Pipetting Needle and the Exposure Capillary Pod is connected *via* optical fibres to a light source and to a spectrophotometer acquisition module. Remote commands allow measurement of the intensity of the light transmitted through the solution (typically at 280 nm), thus enabling determination of the sample concentration.

The Sample Changer can realise the transfer of a given volume of solution from one well to another. If the destination well already contains solution then the depth of liquid inside is detected. To improve mixing of two liquids, the needle moves up through the liquid in the destination well while dispensing the solution. Thorough mixing can be programed, typically after a transfer, by pipetting a small volume into the needle and back to the well a requested number of times.

## 6. Beamline integration of the Sample Changer   

A prototype machine used to test the design, which could store up to eight samples in standard PCR tubes of 0.2 ml volume and three buffers in Eppendorf tubes of 1.5 ml volume, was installed on the ESRF ID14-3 beamline (Pernot *et al.*, 2010 ▶). Two machines were built following the final design. One was installed on the ESRF ID14-3 beamline and later moved to the BM29 beamline. The system was briefly described from a user point of view in the context of the capabilities of the BioSAXS beamline (Pernot *et al.*, 2013 ▶), but lacked the full technical description present in this paper. The second machine was installed on the EMBL-Hamburg PETRA-III P12 beamline. The Sample Exposure Unit is physically installed in the X-ray beam, after a fast beam shutter, beam-definition slits and beam-cleaning slits, with the latter being very important to cut off parasitic scattering coming from the edges of the beam-definition slits. Two fast vacuum valves placed upstream and downstream of the Sample Exposure Unit allow an Exposure Pod to be changed without the need to break the vacuum in the other beamline sections. When the vacuum drops below a threshold value, a vacuum sensor immediately closes both valves, thus preventing contamination of the upstream beamline and the downstream flight tube. Specific beamline hardware controls pumping and venting of the Sample Exposure Cell. Two electrical signals are connected to the control unit of the Sample Changer (§[Sec sec3]3) to ensure safe operation of the beamline. ‘Pod in place’ indicates that vacuum can be applied to the Sample Exposure Unit; ‘Vacuum Is OK’ indicates that an experiment can be performed. In the case of a capillary leak the ‘Vacuum Is OK’ signal becomes false and capillary filling and cleaning are stopped.

Software integration of the robot is simplified by the use of a dynamic server that allows the communication protocol most appropriate to the beamline to be chosen. The software defines a server interface (a set of methods to be exported) and dynamically creates a server to publish this interface. The server type and parameters are defined in the configuration dialogue in the GUI. Many server types are supported: standard protocols (Web Services, RMI), common synchrotron-control systems (Epics, Tango and Tine) and also EMBL’s own protocol called Exporter, having client libraries in Java, Python and C. The Exporter protocol is very convenient for Java clients, which can access the server interface through a dynamic proxy and also can receive asynchronous events from the server to speed up its updating.

Customization is easy thanks to the ability to override hardcoded canonical operation sequences with scripts written in Python. In this way all robot tasks can be modified to conform to local requirements. Furthermore, the application can be customized with plugins in order to add GUI features, new devices or to extend the server interface.

Tango and Tine device servers are used to integrate the sample changer at the ESRF and EMBL@PETRA-III beamlines, respectively.

The Sample Changer functions can be triggered manually or automatically by the data-acquisition software *BsxCuBE* (Pernot *et al.*, 2013 ▶) at the ESRF and *Becquerel* (Franke *et al.*, 2012 ▶) at EMBL-Hamburg.

## 7. Performance and use of the Sample Changer   

Samples and buffer solutions can be stored in the Sample Storage Rack between 4 and 40°C. 5–250 µl of solution can be automatically loaded and exposed at a temperature between 4 and 60°C in both static or flow mode. The pods are available in two tubing lengths: short for fast transfer (50 µl maximal volume loaded) or long for larger volumes (up to 250 µl). The typical sample-turnover time is 50 s, which allows a concentration series of three protein measurements and the matched buffer backgrounds to be performed in about 5–10 min depending on the volume to be loaded, the exposure time, the viscosity and the temperature difference between the Sample Storage Rack and the Sample Exposure Units. The turnover time includes 10–20 s needed to load the solution into the Exposure Cell, 10–20 s to recover the sample (optional) and 8–20 s to wash, rinse and dry the Exposure Cell and tubing. The viscosity can be set to low, medium or high. Each value has predefined default parameters for loading speed, washing and rinsing times, which can be adapted for each installation site (although so far this has not been necessary). The definitions of viscosity for sample loading are (i) low, less than 1.3 mPa s (10% glycerol), (ii) medium, between 1.3 and 1.8 mPa s (10–20% glycerol), and (iii) high, above 1.8 mPa s (20% glycerol). As viscosity increases further sample losses will increase owing to material remaining on the surface of the tubes. These values are indicative and will vary depending on other additives and the samples in question present in solution. If necessary, higher viscosity settings can be used if there is suspicion of sample loss during loading that one wishes to reduce or if contamination persists after cleaning (the buffer before and after the sample do not match). A ‘super’ cleaning protocol can be used when samples which are particularly radiation-sensitive leave deposits on the walls of the capillary after normal cleaning. Concentrated cleaning solution stored in an Eppendorf tube is loaded using the Load Sample function and held in place in the capillary using the ‘Fix Sample Position’ subroutine and/or moved back and forth using the sample-position control arrows in the GUI (Fig. 7 ▶) through the capillary to allow it to act on the deposits efficiently. This cleaning action together with the heating of the Sample Exposure Cell to 50°C gives excellent results.

**Figure 8:**
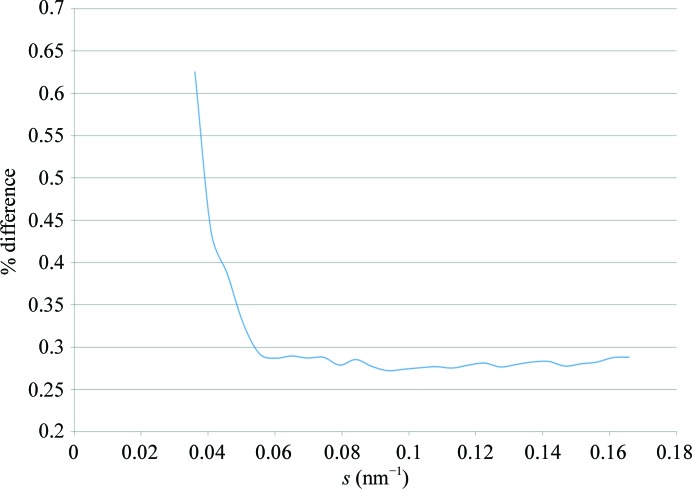
Percentage variation of the background signal caused by deposition on the capillary surface. This plot takes the maximum observed variation in scattering intensity of the daily water-calibration measurements over a period of one month compared (as a percentage difference) with the scattering [*s* = 4πsin(θ)/λ] of a standard protein (calibration measurement of bovine serum albumin at a concentration of around 5 mg ml^−1^). Above *s* = 0.06 nm^−1^ the maximum variation attributed to contamination of the capillary is less than 0.3%. Below *s* = 0.06 nm^−1^ the effect of contamination rises to 0.6%, but as this signal will also be present in the background and thus accounted for in the subtraction, the actual effect on the data will be lower.

The Capillary Pods are easily replaced, allowing the installation of a new clean capillary whenever necessary. However, the automated cleaning of the cells has greatly surpassed expectations by maintaining the same background for several months (see Fig. 8 ▶ for details). Also, the operation of the Sample Changer was proven to be highly reliable: at the EMBL@PETRA-III P12 beamline over 80 000 samples were loaded and measured during 2013, with only five failures owing to various reasons, corresponding to a failure rate of below 0.01%.

## 8. Conclusions   

The BioSAXS Sample Changer and the host beamline work together to provide a reliable robust user-friendly platform for high-throughput measurements of samples in solution. The BioSAXS Sample Changer has been in continual operation (with minimal downtime and maintenance) at the ESRF since 2010 and at P12 at PETRA III in Hamburg since it opened to users in 2012. Users have come to rely on the Sample Changer, with many projects looking at increasingly subtle differences which would not be possible without the reliability of the cleaning process and the stability of the beamlines in general. With achievable cycle times of less than 1 min, measurement of several hundred samples is easily achievable with this setup. Together with the low sample consumption (as little as 5 µl per measurement) the system exploits the brilliant beams available at modern third-generation synchrotron SAXS beamlines in a highly efficient manner.

Manually defining experiments which take full advantage of the capacity of the Sample Changer quickly becomes error-prone. Additionally, the large amount of data from automatic measurements requires curation. It is for these reasons that the ISPyB database has been extended for BioSAXS, with the GUI designed to facilitate the definitions of samples in 96-well plates (De Maria Antolinos *et al.*, 2015 ▶).

Having helped with a large number of projects, there are now a considerable number of publications from beamlines using the BioSAXS Sample Changer. Some examples of the experiments possible using the Sample Changer are given in the beamline paper for ESRF BM29 (Pernot *et al.*, 2013 ▶). Verification of which of several known structures is the physiologically relevant form in solution (Santiago *et al.*, 2009 ▶), can be accomplished with a single concentration series taking approximately 5 min with the sample changer. Studies looking for very subtle differences such as differentiating between cis and trans binding forms (Bahlawane *et al.*, 2010 ▶), where the difference in the radius of gyration is less than 0.2 nm are enabled by the reliability and reproducibility of the cleaning. Functional studies of active complexes under multiple conditions to investigate conformational changes during function (Zerrad *et al.*, 2011 ▶), requiring the measurement of a large number of samples under stringent conditions, are possible due to the reliability and high throughput capabilities of the sample changer. Interdisciplinary collaborations such as those performed as part of the joint SAXS/SANS (facilitated by the PSB SAS platform) which using the combination of many complementary techniques (Lapinaite *et al.*, 2013 ▶), are helped by the easy-to-use interface of the sample changer. Online SEC coupled operation (Round *et al.*, 2013 ▶), benefits from measurements using the Sample Changer as well as SEC, and thus the rapid automated switching between both systems greatly improves the beamtime efficiency. At the EMBL-Hamburg beamlines, the use of the Sample Changer has led to numerous exciting results in the studies of various macromolecular solutions. The sample changer is particularly helpful for projects that require the measurement of large numbers of samples under varying conditions. An example can be found in Sergeeva *et al.* (2014 ▶), where the authors studied the solution properties of several tailor-made polyethylene glycol polymers with antioxidant moieties at different concentrations and temperatures. The high-throughput and extensive screening possibilities offered by the Sample Changer permitted full automation of the measurements and the rapid extraction of phase diagrams of the polymers. The PEG molecular weight is found to influence the lower critical solution temperature, and the results provide a solid basis for the creation of thermosensitive polymers. These measurements also revealed that the customizable flow mode implemented in the Sample Changer is extremely useful to limit the radiation damage that occurs in most samples with the highly intense beam at P12 (Jeffries *et al.*, 2015 ▶).

Overall, the BioSAXS Sample Changer has proved to be highly efficient in offering reliable high-throughput operation for the rapidly increasing user turnover and increasingly demanding research experiments performed on modern high-brilliance synchrotron X-ray beamlines.
